# Session-RPE Method for Training Load Monitoring: Validity, Ecological Usefulness, and Influencing Factors

**DOI:** 10.3389/fnins.2017.00612

**Published:** 2017-11-02

**Authors:** Monoem Haddad, Georgios Stylianides, Leo Djaoui, Alexandre Dellal, Karim Chamari

**Affiliations:** ^1^Sport Science Program, College of Arts and Sciences, Qatar University, Doha, Qatar; ^2^Exercise Science Program, Health Professions, Lebanon Valley College, Annville, PA, United States; ^3^Inter-University Laboratory of Human Movement Biology, University of Lyon, University Claude Bernard Lyon1, Lyon, France; ^4^FIFA Medical Centre of Excellence, Centre Orthopédique Santy, Lyon, France; ^5^Athlete Health and Performance Research Centre, ASPETAR, Qatar Orthopaedic and Sports Medicine Hospital, Doha, Qatar

**Keywords:** perceived exertion, training sessions, competitions, individual sports, team sports

## Abstract

**Purpose:** The aim of this review is to (1) retrieve all data validating the Session-rating of perceived exertion (RPE)-method using various criteria, (2) highlight the rationale of this method and its ecological usefulness, and (3) describe factors that can alter RPE and users of this method should take into consideration.

**Method:** Search engines such as SPORTDiscus, PubMed, and Google Scholar databases in the English language between 2001 and 2016 were consulted for the validity and usefulness of the session-RPE method. Studies were considered for further analysis when they used the session-RPE method proposed by Foster et al. in 2001. Participants were athletes of any gender, age, or level of competition. Studies using languages other than English were excluded in the analysis of the validity and reliability of the session-RPE method. Other studies were examined to explain the rationale of the session-RPE method and the origin of RPE.

**Results:** A total of 950 studies cited the Foster et al. study that proposed the session RPE-method. 36 studies have examined the validity and reliability of this proposed method using the modified CR-10.

**Conclusion:** These studies confirmed the validity and good reliability and internal consistency of session-RPE method in several sports and physical activities with men and women of different age categories (children, adolescents, and adults) among various expertise levels. This method could be used as “standing alone” method for training load (TL) monitoring purposes though some recommend to combine it with other physiological parameters as heart rate.

## Introduction

Using a valid and reliable practical tool is imperative for monitoring the training load (TL) imposed on the athlete during every training session. That would assure the optimal adaptation to training before the competition and also reduce the risks of overtraining. Several methods have been proposed to monitor the TL. Foster et al. (2001) proposed a method based on Rating of Perceived Exertion (RPE). This method, known as session-RPE method, takes into consideration both the intensity and the duration of a training session. The present review is proposed to assure a best understating of this method and its usefulness for monitoring training in athletes. Therefore, the objective of this review is to (1) Retrieve the literature validating the Session-rating of RPE-method using various criteria, (2) Highlight the rationale of this method and its ecological usefulness, and (3) Describe factors that can alter RPE that end-users of this method should take into consideration.

Search engines such as SPORTDiscus, PubMed, and Google Scholar databases, for the time period ranging between 2001 and 2016 were consulted for the validity and usefulness of the session-RPE method. Studies were considered for further analysis when they used the session-RPE method proposed by Foster et al. (2001). Participants were athletes of any gender, age, or level of competition. Studies using languages other than English were excluded in the analysis of the validity and reliability of the session-RPE method. Other studies were examined to explain the rationale of the session-RPE method and the origin of RPE.

## Session-RPE method: rationale

The session-RPE method takes into consideration the intensity and the duration of the training session (or competition) to calculate the TL or competition load. The session-duration refers to the length of session expressed in minutes. A nominal score is given by an athlete to describe his RPE of “mean training intensity” during that training session or competition. Indeed, this method is not only valid for assessing the load relative to training sessions, but also to competition (with some practical issues for some competitions where it is not easy to set the exposure, e.g., team sports where changes are not limited, and therefore, each player's exposure is not easy to assess). For practicality reasons “Training Load” is used in the rest of the paper, but being aware that this is quite reductive as competition is also included.

Basically, the athlete should answer a simple question: “How was your workout?” using the RPE scale. Foster et al. (2001) have modified the verbal anchors used in the CR-10 scale (Borg, 1962) to reflect the American idiomatic English (e.g., light becomes easy; strong or severe becomes hard). According to Foster et al. (2001) rating 6, 8, 9 is not expressed. This single number provided retrospectively by the athlete, refers to the mean intensity of the entire exercise session. Table 1 presents the CR-10 modified by Foster et al. (2001). The athlete should be familiarized with this scale according to standard procedures (Foster et al., 2001) before beginning to collect reliable measures. The origin of the RPE is explained in the following paragraph.

**Table 1:** The modified CR-10 scale by Foster et al. (2001).

**Rating**	**Descriptor**
0	Rest
1	Very, Very Easy
2	Easy
3	Moderate
4	Somewhat Hard
5	Hard
6	
7	Very Hard
8	
9	
10	Maximal

A single arbitrary unit representing the magnitude of global TL for each session is then calculated by the multiplication of training intensity and the length of training (mins). Example: for a session of 87 min, with RPE of 4 (quite hard), the calculations: 87 × 4 will provide a TL of 348 A.U. (Arbitrary Units).

$$\begin{array}{l} \text{TL}\left(\text{A}.\text{U}.\right)=\text{RPE x session duration }\left(\text{min}\right). \end{array}$$

Where: TL is Training Load; A.U. is Arbitrary Units; RPE is Rating of RPE.

## RPE: origin and elucidation

Since the late 1950's, the concept of RPE in sport and exercise science (also known as the sense of effort or perception of effort) has been the subject of increasing thoughtfulness in the scientific literature (Borg, 1962). It has been defined as the conscious sensation of how hard, heavy, and strenuous a physical work is. Its neurophysiological bases are poorly understood despite its importance and usefulness to monitor and prescribe exercise intensity. Some physiologists investigating central regulation of exercise (Proske, 2005; Dempsey et al., 2008) proposed a popular model in which the sense of effort results from the complex integration of different inputs to the central nervous system. Afferent feedback from the peripheral organs (i.e., skeletal muscles, heart, and lungs) and other interceptors (e.g., knowledge of the exercise work endpoint) might be examples of these inputs. Scientists are still continuing their investigations and debates (back >150 years) about the origin of RPE and its dependency on efferent and/or afferent sensory signals. Some researchers proposed a corollary discharge model of RPE where the sense of effort (somatosensory areas) is independent from the skeletal muscles, heart, and lung responses. This model confirms that the narrower definition of exertion as “the effort expended in performing a physical activity” (Oxford Dictionary of Sports Science and Medicine) is physiologically suitable. This might be a good explanation of the verbal descriptors chosen by Borg in 1962 and then Foster in 2001 for their RPE scales (Borg, 1962; Foster et al., 2001). Actually, they used “heavy/hard” and “light/easy” rather than “pleasant/unpleasant,” “feeling good/ feeling bad,” “comfortable/uncomfortable”) as ratings of hedonistic (Marcora, 1985). This proposal was confirmed by a sophisticated experiment conducted by Pollak et al. (2014). The results of the latter study showed that metabolite concentrations injected into skeletal muscle in rested state evoked pain-unrelated (e.g., pressure-, movement-, and thermal-related sensations) and pain-related (i.e., ache) sensations. These concentrations of metabolites stimulated muscle afferents similar to what have been shown during exercise, while the participants reported no perception of effort. It is then clear that injection of metabolites into skeletal muscle at rest does not generate an increase of effort sensation which is the most important feature of fatigue occurring throughout exercise (Barry and Enoka, 2007). This conclusion is in-line with some previous studies (Hamilton et al., 1985; Marcora, 1985) showing that humans are able to distinguish between sensations of pain and sensation of effort during exercise.

## Session-RPE method: derived simple calculations

The “monotony” and “strain” indexes can also be calculated from session-RPE method data of a training microcycle. Actually, the training monotony is a measure of day-to-day training variability that has been found to be related to the onset of overtraining when monotonous training is combined with high TLs (Foster, 1998). Training monotony is calculated using the following formula:

$$\begin{array}{l} \text{Monotony}=\text{weekly mean TL}/\text{SD} \end{array}$$

Where weekly mean TL is the average daily TL during the week and SD is the standard deviation of the daily TL calculated over a week.

Another score that might be a useful unit for monitoring training when players are undertaking high TLs is the training strain. This variable is equal to the multiplication of the weekly TL and the monotony scores. Recovery only becomes fundamental to training when high TLs are being undertaken therefore the calculation of training strain appears advantageous. For example, when TLs are high and there has been low variability of load (resulting in a high monotony index), the training strain is high. This type of training has been associated with incidence of illness and poor performance (Putlur et al., 2004). Conversely, training strain is low when players complete either high or low TLs with regular variation in load sessions (i.e., low monotony). In general, high levels of training strain are usually only reached during the preparation period of training when there is no regular competition.

$$\begin{array}{l} \text{Training strain}=\text{Weekly TL x monotony} \end{array}$$

Spreadsheets can be used to facilitate the calculation of these scores within team and individual athletes. These scores are quite important to allow an optimal periodization of the training as described below.

## Session-RPE method: validity and reliability

With the development of the microtechnologies, many devices are henceforth widely used in the sport industry to monitor TL. Heart rate (HR) monitors, global positioning systems (GPS), accelerometers and wearable body metrics might be some examples of these microtechnologies that can provide coaches with very detailed information on the external (e.g., distance, steps, speed) and internal (e.g., HR, real time electrocardiography, HR variability) TL related variables to be quantified in training sessions.

Although they have the ability to track precise information, these devices have several limitations such as expensive cost, the requirement of high technical expertise and the risk of losing data due to technical error. In addition, these data are quite complex and most of all, to the best of the authors' knowledge, no method has yet worked on getting one single value for a training session TL.

The session-RPE method seems to be a very interesting solution and it has been proposed as a simple, non-invasive and inexpensive method for monitoring TL. The use of the session-RPE method as a practical tool is led by its strong correlations with selected objective methods described above in different types of training.

Actually, the session-RPE method was mainly proposed by Foster et al. (2001) as a simple system to monitor TL of several training modalities. Since 2001 to December, 17th, 2016, this method has been used in 950 studies (PubMed, SPORTDiscus and Google Scholar Search). Most of these studies focused on the validity of the session-RPE method during several technical and tactical sessions of individuals (Minganti et al., 2010; Haddad et al., 2011a,b, 2013a, 2014a; Rodriguez-Marroyo et al., 2012; Tabben et al., 2013; Padulo et al., 2014), team (Manzi et al., 2010; Casamichana et al., 2013), aquatic (Wallace et al., 2009; Minganti et al., 2011a; Dellavalle and Haas, 2012) sports or in different modalities of strength and conditioning training such as, aerobic (Foster et al., 2001; Haddad et al., 2011a), intermittent (Foster et al., 2001; Haddad et al., 2011a), speed, plyometric, and resistance training (Singh et al., 2007; Alexiou and Coutts, 2008; Lockie et al., 2012) and tests (Herman et al., 2006; Haddad et al., 2011a). Table 2 presents an extensive database containing all studies (36 studies) that have analyzed the validity and reliability of the session-RPE method.

**Table 2:** Studies reporting the Validity and reliability of RPE-based method of determining training load proposed by Foster et al. (2001).

**Studies (Classified following the publication date)**	**Sports/Physical activity**	**Gender**	**Age range**	**Number of participants**	**Numbers of sessions/duration of training**	**Criteria**	**Correlations**	**Significance**	**ICC (95% CI)**	**Variability**
Foster et al., 2001	Steady state and Interval exercise	Males	23.0 ± 3.6	6	15 sessions	Edwards TRIMP	–	*p* < 0.05	–	–
		Females	23.0 ± 3.6	6						
	Basketball	Males	20.2 ± 1.5	14						
Sweet et al., 2004	Resistance training	Males	26.1 ± 10.2	10	–	1 RM	–	*p* < 0.05	–	–
		Females	22.2 ± 1.8	10						
Day et al., 2004	Resistance training	Males	24.7 ± 3.8	9	–	1 RM	–	*p* ≤ 0.05	0.88^*^ (0.70–0.96)‡^*^	–
		Females	22.1 ± 2.6	10						
McGuigan and Foster, 2004	Resistance training	Males	21.6 ± 1.2	8	5 sessions	1 RM		*p* < 0.05	0.95^*^ (0.90–0.97)^*^	–
		Females	20.6 ± 0.9	9		Salivary cortisol		*p* < 0.05		
Impellizzeri et al., 2004	Soccer	Males	17.6 ± 0.7	19	476 individual sessions (7 weeks)	Edwards's TRIMP	*r* = From 0.54 to 0.78	From *p* < 0.01 to *p* < 0.001	–	–
						Banister's TRIMP	*r* = From 0.50 to 0.77			
						Lucia's TRIMP	*r* = From 0.61 to 0.85			
Egan et al., 2006	Different resistance training techniques in squad exercise	Females	22 ± 3	14	3 experimental trials	1 RM	–	*p* ≤ 0.05	–	–
Herman et al., 2006	30-min constant-load exercise bouts	Males	33 ± 16	7	7 sessions	%VO_2peak_		*p* < 0.05	0.78^*^	*R*^2^ = 0.76
		Females	23 ± 1	7		%HR_peak_				*R*^2^ = 0.74
						%HR_reserve_				–
Alexiou and Coutts, 2008	Soccer (All sessions)	Females	19.3 ± 2.0	15	16 weeks	Edwards' TRIMP	*r* = From 0.56 to 0.97	*p* < 0.01	–	–
						Banister's TRIMP	*r* = From 0.67 to 0.95			
						Lucia's TRIMP	*r* = From 0.61 to 0.85			
	Conditioning training sessions				139 sessions	Edwards' TRIMP				
					139 sessions	Banister's TRIMP	*r* = 74			
					119 sessions	Lucia's TRIMP	*r* = 60			
	Matches				65 sessions	Edwards' TRIMP	*r* = 64			
					65 sessions	Banister's TRIMP	*r* = 49			
					56 sessions	Lucia's TRIMP	*r* = 49			
	Speed training sessions				59 sessions	Edwards' TRIMP	*r* = 79			
					59 sessions	Banister's TRIMP	*r* = 61			
					48 sessions	Lucia's TRIMP	*r* = 75			
	Technical training sessions				230 sessions	Edwards' TRIMP	*r* = 82			
					230 sessions	Banister's TRIMP	*r* = 68			
					200 sessions	Lucia's TRIMP	*r* = 69			
	resistance training sessions				242 sessions	Edwards' TRIMP	*r* = 0.52			
					242 sessions	Banister's TRIMP	*r* = 0.25			
					200 sessions	Lucia's TRIMP	*r* = 0.34			
Singh et al., 2007	Different types of resistance training	Males	26.7 ± 4.3	15	5 exercises	1 RM	–	*p* < 0.05	–	–
Borresen and Lambert, 2008	Physical activity	Males	–	15	2 weeks	Edwards' TRIMP	*r* = 0.84	*p* < 0.05	–	–
		Females		18		Banister's TRIMP	*r* = 0.76			
Wallace et al., 2009	Swimming		22.3 ± 3.1		248 individual sessions	Distance	*r* = From 0.37 to 0.85	*p* < 0.01	–	–
		Males		6		Edwards' TRIMP	*r* = From 0.63 to 0.91			
						Banister's TRIMP	*r* = From 0.55 to 0.92			
		Females		6		Lucia's TRIMP	*r* = From 0.59 to 0.94			
						Session-RPE coach	*r* = From 0.73 to 0.94			
Minganti et al., 2010	TeamGym	Females	21.7 ± 1.2	10	3 120-min experimental training sessions	Edwards' TRIMP	*r* = From 0.77 to 0.85	*p* < 0.01	–	*R*^2^ = From 0.59 to 0.85
	Trampette						*r* = 0.90			*R*^2^ = 0.81
	Floor						*r* = 0.77			*R*^2^ = 0.59
	Tumbling						*r* = 0.92			*R*^2^ = 0.85
Manzi et al., 2010	Basketball	Males	28 ± 3.6	25	200 sessions (12 weeks)	Edwards' TRIMP	*r* = From 0.77 to 0.85	From *p* < 0.01 to *p* < 0.001	0.95^*^	–
						Banister's TRIMP	*r* = From 0.70 to 0.82			
Haddad et al., 2011a	Taekwondo	Males	13.1 ± 2.4	10	308 individual sessions (2 weeks)	Edwards' TRIMP	*r* = From 0.56 to 0.86	*p* < 0.001	–	–
						Banister's TRIMP	*r* = From 0.56 to 0.90			
	Aerobic training				107 individual sessions	Edwards' TRIMP	*r* = 0.57	*p* < 0.001		
						Banister's TRIMP	*r* = 0.60			
	Technique and Tactique of Taekwondo				105 individual sessions	Edwards TRIMP	*r* = 0.61	*p* < 0.001		
						Banister's TRIMP	*r* = 0.60			
	Intermittent Exercises/Plyometric Training/Speed				72 individual sessions	Edwards' TRIMP	*r* = 0.31	*p* < 0.01		
						Banister's TRIMP	*r* = 0.32			
Perandini et al., 2011	Taekwondo	Males	23.7 □± 2.2	7	22 sessions	Edwards TRIMP	*r* = 0.64	*p* < 0.01	–	–
						Banister's TRIMP	*r* = 0.52	p = 0.02		
		Females	18.8 ± 1.5	4						
						Lucia's TRIMP	*r* = 0.71	*p* < 0.01		
Minganti et al., 2011a	Diving	Males	25.7 ± 6.1	3	6 sessions (1 week)	Edwards' TRIMP	*r* = 0.81	*p* < 0.01	–	*R*^2^ = 0.66
						Total number of dives	*r* = 0.18	p > 0.05 n.s.		–
		Females	25.3 ± 0.6	3						
						Total degree of difficulty	*r* = 0.14	p > 0.05 n.s.		–
						Average degree of difficulty	*r* = 0.75	*p* < 0.01		*R*^2^ = 0.56
Haddad et al., 2011b	Intermittent training in Taekwondo	Males	14 ± 2	18	1 week	Edwards' TRIMP	*r* = 0.20	p > 0.05 n.s.	–	–
						Banister's TRIMP	*r* = 0.14			
Gomez-Piriz et al., 2011	Soccer (Small-sided games)	Males	26.74 ± 4.2	23	13 sessions	Total body load	β = 0.23	*p* < 0.05	–	–
Minganti et al., 2011b	Interval training (rest periods are taken into account)	–	45.3 ± 7.3	18		Edwards' TRIMP	*r* = 0.82	p = 0.013	–	*R*^2^ = 0.67
	Interval training (rest periods were eliminated for the session-RPE computation)						*r* = 0.86	p = 0.003	–	*R*^2^ = 0.74
Moreira et al., 2012	Official Basketball matches	Males	26.4 ± 3.8	10	2 matches	Salivary Cortisol	*r* = 0.75	*p* < 0.001	–	–
Akubat et al., 2012	Soccer	Males	17 ± 1	9	6 weeks	Banister's TRIMP	*r* = 0.75	p = 0.02	–	
						% Δ vLT	*r* = 0.13	p > 0.05 n.s.		–
						% Δ vOBLA	*r* = 0.40			
						% Δ LT_HR_	*r* = 0.20			
						% Δ OBLA_HR_	*r* = 0.15			
Rodriguez-Marroyo et al., 2012	Cycling	Males	25 ± 1	12	2 consecutive seasons	Lucia's TRIMP	*r* = 0.75	*p* < 0.001	–	–
Dellavalle and Haas, 2012	Rowing	Males	19.7 ± 0.8	7	64 sessions	Edwards TRIMP	*r* = 0.88	*p* < 0.001	–	*R*^2^ = 0.77
Casamichana et al., 2013	Soccer	Males	22.9 ± 4.2	28	44 sessions	Total distance covered	*r* = 0.74	*p* < 0.01	–	–
						Player load (determined by accelerometer)	*r* = 0.76			
					220 individual sessions	Frequency of efforts at high speed	*r* = 0.64			
						Work:rest ratio	*r* = 0.29			
						Edwards TL	*r* = 0.57			
Scott et al., 2013	Australian Football	Males	19.0 ± 1.8	21	38 sessions (13 weeks)	Edwards' TRIMP	*r* = 0.83	*p* < 0.05	–	–
						Banister's TRIMP	*r* = 0.83			
						% HR peak	*r* = 0.66			
						Total distance covered	*r* = 0.81			
						Player load (determined by accelerometer)	*r* = 0.83			
						High speed running	*r* = 0.71			
	8 min at speeds of 10 km × h^−1^ (Level 1)		16.1 ± 0.5	10	Twice a week				0.55¥	–
	8 min at speeds of 11.5 km × h^−1^(Level 2)				Twice a week				0.55¥	
	8 min at speeds of 13 km × h-1(Level 3)				Twice a week				0.79^*^	
Clarke et al., 2013	Canadian Football	Males	22.0 ± 1.4	12	713 individual sessions (11 weeks)	Edwards' TRIMP	*r* = From 0.69 to 0.91	*p* < 0.01	–	–
						Banister's TRIMP	*r* = From 0.65 to 0.90			
Lovell et al., 2013	Rugby	Males	24.4 ± 4.1	32	2400 individual training sessions (43 weeks)	Distance	*r* = 0.83	*p* < 0.05	–	–
						High-speed running	*r* = 0.60			
						Body load	*r* = 0.56			
						Impacts	*r* = 0.55			
						Banister's TRIMP	*r* = 0.75			
	Conditioning			22	15.3 ± 2.9 sessions	Distance	*r* = 0.80 ± 0.11	–	–	–
						High-speed running	*r* = −0.23 ± 0.20			
						Body load	*r* = 0.63 ± 0.15			
						Impacts	*r* = 0.69 ± 0.14			
						Banister's TRIMP	*r* = 0.68 ± 0.19			
	Skills			32	34.3 ± 13.0 sessions	Distance	*r* = 0.69 ± 0.10	–	–	–
						High-speed running	*r* = 0.53 ± 0.15			
						Body load	*r* = 0.51 ± 0.17			
						Impacts	*r* = 0.57 ± 0.11			
						Banister's TRIMP	*r* = 0.45 ± 0.16			
	Skills conditioning			22	13.7 ± 2.2 sessions	Distance	*r* = 0.88 ± 0.05	–	–	–
						High-speed running	*r* = 0.84 ± 0.08			
						Body load	*r* = 0.64 ± 0.14			
						Impacts	*r* = 0.75 ± 0.16			
						Banister's TRIMP	*r* = 0.75 ± 0.23			
	Speed			12	10.8 ± 1.0 sessions	Distance	*r* = 0.79 ± 0.12	–	–	–
						High-speed running	*r* = 0.43 ± 0.15			
						Body load	*r* = 0.58 ± 0.19			
						Impacts	*r* = 0.73 ± 0.15			
						Banister's TRIMP	*r* = 0.56 ± 0.29			
	Wrestling			13	12.2 ± 1.4 sessions	Distance	*r* = 0.37 ± 0.19	–	–	–
						High-speed running	*r* = 0.16 ± 0.21			
						Body load	*r* = 0.45 ± 0.22			
						Impacts	*r* = 0.30 ± 0.22			
						Banister's TRIMP	*r* = 0.56 ± 0.20			
Wallace et al., 2014	Steady-state and interval training sessions	Males	23.8 ± 8.4	5	18 individual sessions (6 weeks)	VO_2_	*r* = 0.75 ± 0.11	*p* < 0.05	0.69‡ (0.53–0.80)¥‡^*^ 0.73‡ (0.58–0.83)¥‡^*^	–
		Females		5		%VO_2max_	*r* = 0.80 ± 0.06			
Borges et al., 2014	Sprint kayak	Males	17.1 ± 1.2	6	7 weeks	Banister's TRIMP Banister's	0.62 6 0.15	*p* < 0.05	–	–
		Females		4						
						iTRIMP				
Padulo et al., 2014	Karate	Males	12.5 ± 1.84	11	10 sessions (1week)	Banister's TRIMP	*r* = from 0.84 to 0.92	*p* < 0.001	–	–
						Edwards' TRIMP	*r* = 0.84 to 0.97			
Lupo et al., 2014	Water polo	Males	12.6 ± 0.5	13	8 sessions/80 individual sessions (10 days)	Edwards' TRIMP	*r* = 0.88	*p* < 0.001	–	*R*^2^ = 0.79
Tabben et al., 2015	Karate	Males	24.2 ± 2.3	10	8 sessions	Banister's TRIMP	*r* = 0.65-0.95	*p* < 0.001	0.81^*^	–
		Females	22.6 ± 1.2	8		Edwards' TRIMP				
Tran et al., 2015	Rowing	Males	25.9 ± 3.4	12	17.4 ± 2.3 individual sessions (4 weeks)	T2 min	*r* = 0.42 ± 0.41	*p* < 0.05	–	–
		Females	29.0 ± 5.7	2						
Rodriguez-Marroyo and Antonan, 2015	Soccer	Males	11.4 ± 0.5	12	20 sessions	Edwards' TRIMP	*r* = 17	p = 0.335 n.s.	–	–
Gaudino et al., 2015	Soccer	Males	26 ± 6	22	1892 individual sessions (38 weeks: entire in-season competitive period)	High-speed-running (>14.4 km/h)	*r* = 0.11	*p* < 0.001	–	–
						Distance and number of impacts	*r* = 0.45			
						Accelerations >3 m/s^2^	*r* = 0.37			
Gomes et al., 2015	Tennis	–	18.4 ± 0.4	12	384 sessions (7weeks)	Edwards' TRIMP	*r* = 0.74	*p* < 0.05	–	–
					23 simulated matches		*r* = 0.57			
					13 official matches		*r* = 0.99			
Campos-Vazquez et al., 2015	Soccer (Skill drills/Small-sided games)	Males	26.7 ± 4.5	9	Full competitive season	Average HR	*r* = −0.06	–	–	
						>80%HR_max_	*r* = 0.23			
						>90%HR_max_	*r* = 0.11			
						Edwards' TRIMP	*r* = 0.55			
	Soccer (Ball-possession games/technical andtactical exercises)					Average HR	*r* = 0.12			
						>80%HR_max_	*r* = 0.61			
						>90%HR_max_	*r* = 0.62			
						Edwards' TRIMP	*r* = 0.87			
	Soccer (Tactical training)					Average HR	*r* = 0.39			
						>80%HR_max_	*r* = 0.67			
						>90%HR_max_	*r* = 0.68			
						Edwards' TRIMP	*r* = 0.80			
	Soccer (prematch activation)					Average HR	*r* = 0.21			
						>80%HR_max_	*r* = 0.34			
						>90%HR_max_	*r* = 0.38			
						Edwards' TRIMP	*r* = 0.50			
de Andrade Nogueira et al., 2016	Swimming	Males	15.8 ± 0.87	10	18 sessions	Total volume	*r* = 0.71	*p* < 0.05	–	–
						Aerobic volume	*r* = 0.58			
						High intensity volume	*r* = 0.45			
		Females	15.1 ± 0.46	7						
						Severe intensity volume	*r* = 0.43			
						Anaerobic volume	*r* = 0.35			
Turner et al., 2017	Fencing	Males	21.8 ± 2.3	7	67 sessions	Banister's TRIMP	*r* = 0.84 − 0.98	*p* < 0.05	0.55¥	–
						Edwards' TRIMP	*r* = 0.91 − 0.98			
					101 competition bouts	Banister's TRIMP	*r* = 0.89 − 0.99			
						Edwards' TRIMP	*r* = 0.92 − 0.99			
					67 footwork (training)	Banister's TRIMP	*r* = 0.73	*p* < 0.05		
						Edwards' TRIMP	*r* = 0.79	*p* < 0.01		
					67 sparring (training)	Banister's TRIMP	*r* = 0.76	*p* < 0.05		
						Edwards' TRIMP	*r* = 0.85	*p* < 0.01		
					85 poules (competition)	Banister's TRIMP	*r* = 0.82	*p* < 0.05		
						Edwards' TRIMP	*r* = 0.89	*p* < 0.01		
					12 knockouts (competition)	Banister's TRIMP	*r* = 0.92	*p* < 0.05		
						Edwards' TRIMP	*r* = 0.91	*p* < 0.01		
Lupo et al., 2017	Taekwondo	Males	12.0 ± 0.7	5	pre-competitive and competitive periods	Edwards' TRIMP	*r* = 0.71	*p* < 0.05	–	–
		Females	12.0 ± 0.8	4						

ICC, intraclass correlation coefficient of the RPE-method (<0.40—poor^¤^, Between 0.40 and 0.59—Fair^¥^, Between 0.60 and 0.74^‡^–Good, Between 0.75 and 1.00—Excellent^*^); r = correlation coefficient between RPE-method and various methods used as criterion; p, significance level (p > 0.05, ns) between RPE-method and various methods used as criterion; R^2^, coefficient of determination between RPE-method and various methods used as criterion; CI, confidence interval; RPE, Rating of perceived exertion; RM, Repetition maximum; VO_2_, Oxygen consumption; VO_2max_, Maximal oxygen uptake; HR, Heart rate; TRIMP, Training Impulse; iTRIMP, Individual training impulse; vLT, Velocity at 2 mmol · L^−1^; LTHR, Heart rate at 2 mmol · L^−1^; vOBLA, Velocity at 4 mmol · L^−1^; OBLAHR, Heart rate at 4 mmol · L^−1^; –: Missing Data.

## Session-RPE method: correlations with objective markers

As presented in Table 2, the session-RPE method was correlated with several objective markers of TL. In the following section, mentioned objective markers in Table 2 are defined.

Bannister's training impulse (TRIMP) is a method used to quantify TL. It is calculated with a pre-evaluated coefficient obtained with the relationship between HR and blood lactate during an incremental exercise (y), multiplied with the HR reserve (HRres) and the duration of the sessions (t) (Banister, 1991). Its use was correlated to the session-RPE method during soccer training and match-, swimming-, basketball-, Taekwondo-, Australian football-, Canadian football-, rugby-, kayak sprinting-, karate training and match-, and fencing training and competing-sessions (Table 2).

Edwards' TRIMP is a method used for the calculation of TL with the time spent in five arbitrary HR zones multiplied by arbitrary coefficient (>50–60% x1; >60–70% x2; >70–80% x3; >80–90% x4; >90–100% x5) (Edwards, 1993). This method was not validated with a known physiological response but was used in several studies as a good indicator of TL. Although, its use was correlated to the session-RPE method during soccer training and match-, swimming sessions, female fitness exercises, basketball-, Taekwondo-, diving-, rowing-, Australian football-, Canadian football-, karate-, water polo-, tennis-, and fencing training and competing- sessions.

Lucia's TRIMP is also a method used to measure TL related to the ventilatory thresholds (VT1 and VT2). VT1 corresponds to the anaerobic threshold and VT2 to the respiratory compensation threshold. This method provides three zones: low (<VT1), moderate (VT1–VT2) and high (>VT2), and each zone corresponds to a coefficient: 1, 2, and 3 respectively. TL is calculated with the time spent in each zone multiplied by the corresponding coefficient, and added to each other (Lucia et al., 2003). Its use was correlated to the session-RPE method during soccer-, and swimming-training sessions.

HR is one of the most relevant objective markers of TL. It can be used with many forms to monitor TL and one of them is the percentage of maximal HR (%HR_max_) observed during exercise. Strong and very strong correlations were found between session-RPE method and %HR_max_ during soccer specific training sessions and during Australian football sessions for instance (Table 2).

The oxygen uptake during exercise (VO_2_) and its expression as percentage of the maximal volume of oxygen uptake (%VO_2max_) were both strongly correlated to the session-RPE method for males and females practicing interval training.

Motion characteristics are frequently used to quantify TL, especially in team sport like rugby, Australian football and soccer, but also in individual sport like swimming as it provides individual information about the total distance covered or the distance covered in high speed displacement, for instance, during training and match.

Lactate threshold (LT) determination is one of the most relevant indicators of aerobic performance and training status. LT is where the pace has been raised to a point where Krebs cycle (aerobic energy), in muscle cells, cannot provide sufficient energy anymore. This threshold is usually observed at around 2 mmol.l^−1^. The second threshold, where a second increase in lactate accumulation happens around 4 mmol.l^−1^ is referred to the onset of blood lactate threshold (OBLA). The velocity at LT (vLT) and OBLA (vOBLA) are the pace when running just before exceeding the LT and the OBLA point respectively. Their use was strongly correlated to the session-RPE method during soccer training sessions for instance (Table 2).

Some specific moves could also be assessed to quantify the TL, and each sport has its own related specific characteristics. Some of them were strongly correlated with session-RPE method, especially the number and the frequency of impacts in rugby, and the number and the level of difficulty of dives in diving, for instance (Table 2).

## Session-RPE method: ecological usefulness

The session-RPE method might be used for monitoring one session, weekly blocks (mesocycle) and year-to-year periods (macrocycle) as well. Actually, it is widely recognized that the key of success for most athletes is the carefully periodization of different cycles throughout the training plan. The session-RPE method might provide a useful tool to better control the training periodization through monitoring all type of training sessions (Haddad et al., 2011a). Derived scores (i.e., Monotony and Strain) present useful complements to this method as explained above. Many authors have used the session-RPE method for monitoring training cycles such as Impellizzeri et al. (2004) and Akubat et al. (2012) in male Soccer, Alexiou and Coutts (2008) in female Soccer, Wallace et al. (2009) in Swimming, Manzi et al. (2010) in Basketball, Haddad et al. (2011a) in Taekwondo, Scott et al. (2013) in Rugby, and Padulo (Padulo et al., 2014) in Karate. The session-RPE method has also been used for monitoring entire macrocycles by Clarke et al. (2013) and Lovell et al. (2013) in Canadian football, by Murphy et al. (2014) in tennis, by Brink et al. (2014) in soccer and volleyball de Andrade Nogueira (de Andrade et al., 2014) in Volleyball.

The session-RPE method might allow achieving an appropriate TL periodization, therefore the likelihood of excessive TLs would be reduced. This might consecutively reduce the chances of overtraining or injury. Clarke et al. (2013) suggested that session-RPE method might help optimize physical development while minimizing overtraining, injury and illness across the board by enhancing awareness of individual responses to physical stimulus. For instance, application of the Session-RPE method can not only help to carefully manage the players back to full training but also to provide a valuable tool to begin investigating the relationship between training-load/fatigue and injuries (Chamari et al., 2012, 2013).

However, careful attention should be given to the relationship between prescribed/intended and perceived training. Basically, the coach will ask himself: “How will be the intended workload of the training session I'll prescribe?” using the same scale. As an example, the RPE of the coach is that the session intensity will be moderate which means 3 in the CR-10 scale. Athletes should answer a simple question: “How was your workout?” using this RPE scale after the training session. So, coaches and athletes are using the same scale to quantify the exercise intensity. Few studies have investigated the relation between the RPE of the coach (before the session) and the athlete (after the session). In summary, as one example of individual sports, Stewart and Hopkins (1997) showed a weak relationship between the coaches' prescribed intensity and the individual swimmer's perceived intensity. Wallace et al. (2009) showed that well-trained swimmers perceived their training sessions harder for high-intensity and lower for low-intensity training sessions than what was prescribed by the coach. This difference has been confirmed by other authors in individual sports such as Viveiros et al. (2011) in Judo and Murphy et al. (2014) in Tennis. The weak agreement between planned and perceived training dose of a coach and athlete/player was also observed during an entire season of team sports such Soccer (Brink et al., 2014) and volleyball (de Andrade et al., 2014). Brink et al. (2014) showed that the agreement between coaches' and players' perception of TL in a team sport appeared to be weaker in comparison with individual sports (Stewart and Hopkins, 1997; Wallace et al., 2009; Viveiros et al., 2011; Murphy et al., 2014). A logical explanation would be related to the difficulty for coaches to plan and control the exercise intensity for groups rather than individuals. Some solutions should be discovered in order to solve the inconsistencies between coaches prescribed and players perceived such as: (1) Throughout the scheduled training, coaches should keep in mind the specificity and individual characteristics (physical and psycho-social) that might affect the internal load of each individual player/athlete, (2) Use HR monitors to assist new RPE user to match their perceptions with the intensity of their training (Stagno et al., 2007). Controlling the relation between the scheduled and perceived TL of coaches and players/athletes respectively might improve performance by optimizing the TL periodization and preventing injuries and illnesses.

## Factors influencing perceived exertion

It is well known that physiological and neural determinants do not fully explain the variation of RPE (Morgan, 1973) as other factors influence it as well. For instance, sociological factors such as the presence and the type of a co-actor at the moment of RPE collection in addition to personality factors (extraversion, neuroticism, depression, and anxiety) were shown to affect RPE (Morgan, 1973, 1994). Moreover subjects' characteristics such as gender, age, fitness level, and expertise level, might influence RPE as well. Other influences appear with environmental factors such as: listening to music, image and video watching, feedback and instructions about exercise, RPE scales' variation, hypnosis, environmental temperature, altitude, glycemia, the consumption of pharmacological and/or doping products, caffeine-, energy-, alcohol-, milk-chocolate-drinks, Ramadan fasting, and mobilization of attentional resources (Haddad et al., 2014b). Moreover, Haddad et al. (2014a) have suggested that the time spent at high-intensity and only marginally the session duration influenced the session' RPE. The same group has also shown that the reliability of an RPE-scale' translation in another language (other than English; French in that case) is affected despite keeping good internal consistency (Haddad et al., 2013a). However, the subjects' perception of fatigue, stress, delayed onset muscle soreness, and sleep were not major contributors of RPE- for a 10 min sub-maximal effort during training with non-excessive TLs (Haddad et al., 2013b). This provides further evidence that RPE is not totally independent from efferent or afferent sensory signals. On the other hand, the influence of these factors could potentially be observed during high intensity training or overtraining. This still has to be investigated.

These various factors could somewhat alter the perception of exercise intensity; however, scientific literature supports the validity of RPE as indicator of exercise intensity despite any possible influence of contributing factors as described above. The good reliability and internal consistency of RPE in several sports and physical actives with men and women of different age categories (children, adolescents, and adults) among various expertise levels clearly shows the usefulness of the session-RPE method for efficient TL monitoring purposes (Haddad et al., 2014b).

## Conclusion

Session-RPE method has been shown to be valid, reliable and very useful on the field. Nevertheless, other subjective measures also show high value. Coaches and staff cannot also exclude the possibility of adding to subjective measures, other objective measures (e.g., HR measures, adapted for endurance sports, and/or GPS measures, adapted for team) complementing even more the data obtained from subjective measures. Fine individualization is probably a key to training outcome optimization. In order to assess the status of the athlete before the training session, it would be interesting to complement the use of RPE with a “wellness index” (e.g., Hooper index or other tools). This would allow adapting the coming scheduled training session to the actual status of the athlete of that day in that particular moment.

## Author contributions

MH conceived and designed the structure of the mini-review. All authors collected and analyzed the data. All authors drafted and approved the final version of the manuscript.

### Conflict of interest statement

The authors declare that the research was conducted in the absence of any commercial or financial relationships that could be construed as a potential conflict of interest.
